# Towards a Biopsychological Understanding of Costly Punishment: The Role of Basal Cortisol

**DOI:** 10.1371/journal.pone.0085691

**Published:** 2014-01-08

**Authors:** Stefan Pfattheicher, Johannes Keller

**Affiliations:** Department of Social Psychology, University of Ulm, Ulm, Germany; Hungarian Academy of Sciences, Hungary

## Abstract

Recent findings have documented a negative relation of basal endogenous cortisol and aggression after a provocation (i.e., reactive aggression) in humans. We build on these findings and investigated the relation of endogenous cortisol and reactive aggression in a social dilemma situation, that is, costly punishment of individuals who did not appropriately contribute to a common group project. Specifically, we predicted that basal cortisol is negatively related to costly punishment of uncooperative individuals. In the present study, basal cortisol was assessed prior to a public goods game with the option to punish other group members. In line with previous research on reactive aggression and basal cortisol, we found that basal cortisol was indeed negatively related to costly punishment. The findings are important for understanding costly punishment because this tendency has been documented as a possible basis for the evolution of cooperation.

## Introduction

Extensive research in human and non-human animals has shown that psychological stressors activate the hypothalamic-pituitary-adrenal axis (HPA axis) which regulates the release of cortisol [1]. The activation of the HPA axis and cortisol in turn are related to important cognitive, affective and behavioral tendencies such as general life stress, deficits in cognitive functioning, mood disorders like depression, and food consumption [2]–[5]. In recent years, the relation of cortisol and *aggressive behavior* was the center of attention for many research groups, investigating the relation of endogenous and exogenous cortisol with different forms of aggression [6]–[12]. The present work aims to extend this research by investigating the relation of endogenous cortisol and reactive aggression in a social dilemma situation, that is, costly punishment of individuals who did not appropriately contribute to a group project. Specifically, building on recent findings documenting a negative relation of basal endogenous cortisol and aggression after a provocation [7], [8], [10], [12], we predicted that basal cortisol is negatively related to costly punishment. Investigating the relation of basal cortisol and costly punishment is particularly worthwhile because research on costly punishment in public goods situation has neglected the possible role of basal cortisol regarding costly punishment so far. In this sense, the present research addresses a gap in this field of study in that we analyze an important aspect referring to the psychophysiology of costly punishment.

In the following, we first outline the importance of investigating costly punishment in social dilemma situations, then we show why costly punishment reflects reactive aggression and review literature on the relation of cortisol and aggression while focusing on basal endogenous cortisol and reactive aggression. Finally, we report on a study in which basal cortisol was assessed prior to a social dilemma situation (the public goods game) with the option to costly punish other group members.

### The Tragedy of Social Dilemma Situations

To illustrate a social dilemma situation, imagine a public park in which people like to have barbecues and picnics. In this scenario, every individual benefits from leaving their garbage in the park because taking the garbage home involves some effort. Obviously, the problem is that leaving garbage in the park damages the public good (i.e., the public park). This example demonstrates the inherent tragedy of social dilemma situations [13]. Free-riders who behave uncooperatively (i.e., people leaving their garbage behind) benefit compared to cooperative individuals (i.e., people taking their garbage home), which leads to a reduced benefit of the collective (i.e., a polluted park). That is to say, the collective benefits if all behave cooperatively; however, the problem (i.e., the dilemma) is that each individual benefits from selfish behavior which results in a reduced benefit of the collective. This inherent problem of social dilemma situations results in the question of how free-riding can be avoided, that is, how cooperation can be established.

One prominent solution is to establish a system of costly punishment, that is, the option to invest resources to punish uncooperative interaction partners [14], [15]. In the example above, cooperative people could punish through social disapproval or imposing financial penalties on people who pollute the park [16], [17]. Extensive research has shown that humans indeed punish uncooperative free-riders in anonymous one-shot social dilemma situations, even when it is costly and no direct (material) benefit can be obtained [18], [19]. The punishment of uncooperative free-riders (which has been discussed with reference to the term “costly punishment”) has substantial positive effects on the level of cooperation in public goods situations [18], [19] and therefore represents one structural solution of the free-rider problem [20] ^1^.

### Costly Punishment, Reactive Aggression and Basal Cortisol

A large amount of empirical evidence has shown that punishment is mainly imposed on individuals who behaved uncooperatively and did not appropriately contribute to the public good [18], [19]. Costly punishment is predominantly driven by anger and impulsiveness [19], [24]–[32] on basis of a fairness norm violation by low contributors [33]–[35]. In this sense, costly punishment can be considered as an angry impulsive response to perceived provocation or interpersonal frustration (i.e., uncooperative behavior and a perceived norm violation), which reflects reactive aggression [11], [36]. Reactive aggression has to be distinguished from proactive aggression which is defined as aggression without a prior provocation. This type of aggression is typically executed instrumentally in that it is used to achieve a specific outcome (beyond harming another individual) [36]. In this regard, proactive aggression corresponds to the punishment of *cooperative* individuals termed antisocial punishment, because when punishing cooperative individuals no provocation occurred which may trigger reactive aggression. Accordingly, the instrumental motive reflected in status and dominance concerns can be considered a driving force regarding antisocial punishment [37], [38]. That is to say, it is reasonable to assume that costly punishment of uncooperative individuals reflects reactive aggression (this suggestion was explicitly made by different authors [25], [39], [40]) whereas the punishment of cooperative individuals, that is, antisocial punishment, largely reflects proactive aggression [37].

In the present contribution, we deal with the association of endogenous cortisol and reactive aggression, that is, costly punishment. Crucially, recent findings document a negative relation of basal cortisol and reactive aggression.^2^ For instance, Böhnke and colleagues [7] used the Taylor Aggression Paradigm (TAP) to allow for reactive aggression in their participants. The TAP is a competitive reaction time task in which a provocation (e.g., a blast of noise activated by another, actually fictitious, participant) can be answered with an aggressive response (e.g., a blast of noise). Importantly, Böhnke and colleagues [7] found that basal cortisol levels were negatively related to reactive aggression. This result was successfully replicated in another study of this research group [12]. In a similar vein, Poustka and colleagues [10] observed a negative relation of basal cortisol levels and dispositional reactive aggression in men (but not in women). Feilhauer and colleagues [8] observed the same pattern, dispositional reactive aggression was negatively related to basal cortisol levels in a sample of healthy male adolescents. We want to note that other research, however, found no significant relation between basal cortisol and behavioral reactive aggression (in a sample of 7-year-old children [43]). Moreover, van Bokhoven and colleagues [44] document that highly reactive aggressive school boys possessed higher morning cortisol levels than weakly reactive aggressive school boys. Taken together, there is predominantly supportive empirical evidence for an inverse relation of reactive aggression and basal cortisol in adults. Given that we argue that costly punishment reflects reactive aggression (see also [25], [39], [40]), we assume that basal cortisol and costly punishment are negatively related (in healthy male adults).

The question remains which psychological mechanisms and theoretical explanations can account for an inverse relation of endogenous cortisol and costly punishment (we propose a multiple mediator perspective, as emphasized in recent theorizing about mediation, see [45]–[47]). Feilhauer and colleagues [8] argue that *impulsivity* is reflected in the endocrinological pattern of a low basal cortisol level (see also [48], [49]). This view is for instance supported by other research [50] documenting a negative relation of trait impulsivity and cortisol levels. Similarly, Poustka and colleagues [10] showed a negative relation of impulsivity and basal cortisol in healthy men. The negative association between basal cortisol and impulsivity (i.e., high impulsivity in low cortisol individuals) is relevant with respect to costly punishment because costly punishment reflects an impulsive act [25], [26], [32], [51], [52]. For instance, research on ego-depletion has shown that impulsive behavior emerges more likely when cognitive resources are depleted [53], [54]. Interestingly, Halali and colleagues [26] showed that ego-depletion increased costly punishment. In another study, these authors showed that participants were faster in terms of reaction times when engaging in costly punishment (vs. when not engaging in costly punishment). Faster reactions are typically associated with impulsivity [55]. Thus, findings showing (a) an inverse relation of basal cortisol and impulsivity and (b) that costly punishment is an impulsive act speak to our assumption that costly punishment is inversely related to basal cortisol. That is to say, the relation of basal cortisol and costly punishment can be expected based on the common underlying construct of impulsivity.

The proposed inverse relation between cortisol and costly punishment also corresponds to the theory of optimal arousal [11], [56], [57]. This theoretical approach emphasizes that under-aroused individuals possess a pronounced tendency to engage in impulsive acts in order to seek stimulation (cf. [58], [59]). Van Goozen and colleagues [11], [57] have argued that particularly a relatively low cortisol level reflects the state of under-arousal. From this theoretical basis one can assume that under-arousaled individuals (i.e., individuals with a relatively low cortisol level) seek stimulation which might be possible in the dilemma setting by punishing uncooperative free-riders.

Whereas individuals with a relatively low basal level of cortisol are likely to engage in impulsive actions, the reverse was suggested for individuals with a relatively high basal level of cortisol. That is, high cortisol levels are related to behavioral withdrawal [60]–[65]. On this basis one can assume that individuals with a relatively high basal level of cortisol engage in behavioral withdrawal as reflected in the omission of costly punishment [66]. Thus, impulsivity of individuals with low cortisol levels and behavioral withdrawal of individuals with high cortisol levels is in line with the notion of an inverse relation of basal cortisol and costly punishment.

Another line of research also suggests an inverse relation of basal cortisol and costly punishment. Recently, it was found that individuals with a specific *vigilance* to negative social information, that is, the disposition of prevention-focused self-regulation [67]–[69] was positively related to costly punishment [66]. Interestingly, research by van Honk and colleagues [70], [71] showed that low cortisol levels are related to *vigilant* responses to negative social information (angry faces). Thus, there is empirical evidence documenting that a vigilant orientation relates to low cortisol as well as to costly punishment. On this basis we argue that individuals possessing a relatively low level of cortisol possess a special vigilance to negative social information [70], [71], which in turn is positively related to costly punishment [66]. Accordingly, research on vigilant orientations, costly punishment and cortisol also speaks to a negative relation of basal cortisol and costly punishment.

To conclude, building on empirical findings and theoretical considerations from research on cortisol, reactive aggression, impulsivity, behavioral withdrawal, and vigilance, we assume that basal cortisol is inversely related to costly punishment. In the study reported below we put this assumption to an empirical test. We assessed endogenous cortisol prior to a public goods game with the option to costly punish other group members.

## Study

### Method

#### Ethics statement

The study was approved by the Ethics Commission of the University of Ulm and all participants have given written informed consent prior to the study.

#### Participants

Our study involved one hundred and eighty-two healthy non-smoking male volunteers from a German university (*M*_age_ = 22.0, *SD* = 1.55)^3^.

#### Public goods game

In the public goods game, four players constituted one group [19], [72]. Each player was endowed with 20 money units (MUs; 1 MU was equal to €0.05∼$0.06) and free to choose how many of them to keep and how many to contribute to the public good. Each MU contributed was multiplied by 1.6. Next, each of the players received one fourth of the public good, *independently* of their contribution. Accordingly, it was always in the material self-interest of every individual to keep all MUs privately irrespective of how much the other three subjects contributed. If every group member invested 20 MUs, each subject would earn 0.4×80 = 32 MUs. If one group member engaged in free-riding (e.g., he contributed 0 MUs) and the other three group members still invested their 20 MUs, the free-rider earned 44 MUs (20 MUs already owned plus one fourth of the public good, that is, 24 MUs) and each of the other three group members earned 24 MUs.

Afterwards, each player was given accurate information on the contributions of the other three players and had the option to punish them by investing own MUs (between 0 and 10 for each player) that reduced the selected other players’ payoffs by the factor of three (e.g., the investment of 2 MUs decreased the payoff of another by 6 MUs). Six periods of the public goods game were played under anonymous conditions. All interactions were computer-mediated via z-tree [73], and all decisions were made simultaneously. Participants were told that the group composition changed from period to period so that nobody would play twice with a specific other player to exclude direct reciprocity accounts [74]. Participants were privately paid their earnings (*M* = €5.88∼$7.85, *SD* = 1.55) at the end of the session.

#### Costly punishment

In line with Herrmann and colleagues [72], costly punishment was computed by summarizing the MUs across the six periods that were used by each player for the punishment of other players who contributed *less* than the player him/herself.

#### Endogenous cortisol

Endogenous cortisol was measured via two saliva samples collected in sampling tubes (SaliCap®, IBL International GMBH, Hamburg, Germany) 18 and 12 minutes prior to the start of the public goods game. The first measure was taken before participants read the explanation of the public goods game. All first samples were taken around 2∶45 p.m. (±6 minutes) to minimize diurnal variation in hormone concentrations. Cortisol was analyzed in the endocrinological laboratory at Dresden University, Germany, following well-established standard procedures [75]. Intra- and inter-assay coefficients of variation were below 10%. The two saliva samples were strongly correlated (*r* = .86, *p*<.001) and averaged. Cortisol levels were in the normal range (*M = *9.15 nmol/L, *SD = *5.31).

### Results

#### Preliminary results

In order to give the reader an impression of the contributions to the public good and investments in costly punishment, descriptive statistics are reported first. The mean number of MUs invested in the contribution to the public good across the six periods (maximum 6×20 MUs = 120 MUs) was 78.49 MUs (*SD* = 29.27). The mean number of MUs invested in costly punishment across the six periods was 7.32 MUs (*SD* = 9.94). 75.3% engaged in costly punishment at least once. As in the Study of Fehr and Gächter [19], the contributions to the public good and the investments in costly punishment were significantly positively correlated (*r* = .21, *p*<.01).

#### Main analyses

**Figure 1:**
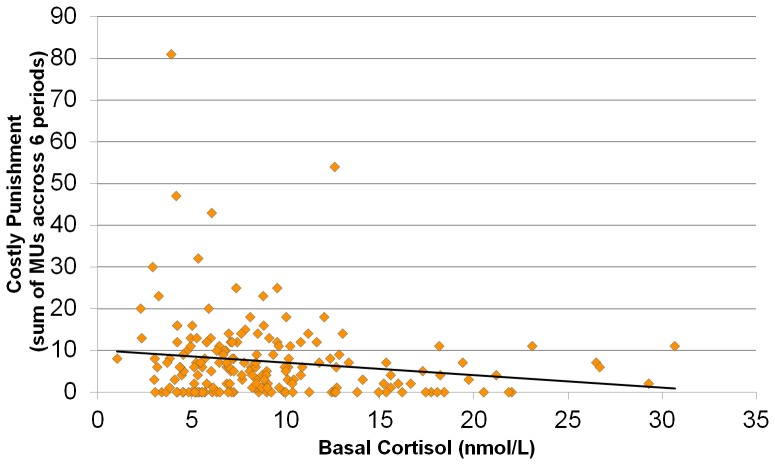
Scatterplot and OLS regression slope of the relation between basal cortisol and costly punishment.

Analyses revealed a significant negative relation between basal cortisol and costly punishment (*r* = −.16, *p*<.05) thus supporting our central assumption (see Figure 1 for a graphical illustration of the relation).

We also tested for over-influential cases, that is, we computed Cook’s distance. The critical value for Cook’s distance is 1 [76], all cases were below.30. We also checked for residual outliers. Three cases were identified. Excluding these cases even strengthen the relation of cortisol and costly punishment. Moreover, bootstrapping this analysis (to apply a non-parametric test) also revealed a significant correlation between cortisol and costly punishment, that is, the 95% confidence interval excluded zero [−0.04, −0.26]. Bootstrapping was based on 5000 re-samples. To account for participants scoring zero on costly punishment (24.7%) we also report Tobit regression [77]: This analysis also revealed a significant relation of basal cortisol and costly punishment (*β* = −.35, *p*<.05). Controlling for the contribution to the public good did not change the coefficients of basal cortisol (partial correlation, *r* = −.16, *p*<.05; Tobit regression: *β* = −.35, *p*<.05). The contribution to the public good was not significantly related to basal cortisol levels (*r* = −.03, *p* = .64).

## Discussion

The specific nature of social dilemma situations results in a potential tragedy [13], [16] because in social dilemma situations individuals benefit from free-riding behavior that damages the benefit of the collective. One structural solution to reduce free riding is to establish a system of costly punishment [20] which substantially increases cooperative behavior [19]. In the present work, we investigated one endocrinological factor that might underlie the punishment of uncooperative free-riders – basal cortisol. Specifically, building on recent findings documenting a negative relation of basal cortisol and aggression after a provocation [7], [8], [10], [12] and the notion that costly punishment reflects reactive aggression [25], [39], [40] we predicted and found that endogenous cortisol is negatively related to costly punishment.

The findings of the present work are important for our understanding of costly punishment of uncooperative individuals because this tendency has been documented as a possible basis for the evolution of cooperation [17], [19], [78], [79]. That is, without having a possibility of punishing uncooperative individuals, cooperation is hardly established in social groups facing a social dilemma situation [19]. Our results suggest that low basal cortisol levels function as a precondition for the engagement in costly punishment. In others words, low basal cortisol levels seem to be adaptive here because they are related to sanctions that foster cooperation in a remarkable way [18], [19]. Therefore, the present work is particularly relevant for our understanding of the evolution of cooperation through costly punishment. Our findings emphasize the notion that it seems crucial to take basal endocrinological factors into account to reach a comprehensive understanding of the evolution of cooperation.

Considering the present contribution, one particular strength is that we applied a well-established paradigm to investigate costly punishment [19] which involves real social behavior, that is, the punishment of the participants involved real monetary costs, real interactions between participants and real effects for the punished individual. Moreover, by measuring endogenous cortisol which is unaffected by socially desirable response tendencies and does not require difficult, frequently biased introspection, we addressed a particularly meaningful interpersonal difference which is specifically relevant from a psychophysiological perspective. Accordingly, we think it is fair to conclude that our research strategy complies with high methodological standards.

The question remains which psychological mechanisms and theoretical explanations can account for the inverse relation of endogenous cortisol and costly punishment. As outlined in the introduction, one possibility is implemented in the basic notion of optimal arousal [11], [56], [57] emphasizing that under-aroused individuals possess a pronounced tendency to engage in impulsive acts [58], [59]. That is to say, under-aroused individuals (i.e., individuals with relatively low cortisol levels) seek stimulation which might be possible by punishing uncooperative free-riders. Whereas low basal level of cortisol are related to impulsive actions (such as costly punishment), high levels of cortisol have been linked to behavioral withdrawal [60]–[65]. Accordingly, individuals with a relatively high basal level of cortisol might show behavioral withdrawal, as could be reflected in the omission of costly punishment [66]. Thus, impulsivity in low cortisol individuals and behavioral withdrawal in high cortisol individuals could simultaneously function as mediators regarding the inverse relation of basal cortisol and costly punishment.

Another approach to the question of why endogenous cortisol and costly punishment are inversely related refers to the concept of vigilance. Research by van Honk and colleagues [70], [71] showed that low cortisol levels are positively related to vigilant responses to negative social information. Recently, Pfattheicher and Keller [66] showed that individuals with a strong disposition of prevention-focused self-regulation, that is, individuals possessing a vigilant sensitivity to negative social information, engaged more likely in costly punishment compared to weakly prevention-focused individuals. Integrating these lines of research, we argue that individuals with relatively low levels of cortisol possess a special vigilance to negative social information. Given that an uncooperative behavior of another group member constitutes negative social information [66], it seems plausible to assume that individuals relatively low (vs. high) in basal cortisol are more likely to engage in costly punishment due to their special vigilance (regarding negative social information).

Recently, Montoya et al. [80] proposed that low levels of endogenous cortisol, high levels of testosterone, and low levels of serotonin together contribute to individuals’ tendency to engage in reactive aggression. It has not only been shown that basal cortisol and testosterone are related to reactive aggression [81]. Also, the basal level of the neurotransmitter serotonin was found to be negatively related to costly punishment [82], and accordingly, the depletion of tryptophan, which lowers brain serotonin levels, increases costly punishment [25]. In the present study, we did not focus on the interaction proposed by Montoya et al. [80]. Rather, we merely included cortisol and focused on the role of this endocrinological factor. This might explain why we found a rather weak relation of cortisol and costly punishment. As proposed by Montoya et al. [80], the relation is likely to be moderated by serotonin and testosterone, and accordingly, a stronger relation of cortisol and costly punishment should emerge when these additional factors are also taken into account, for instance in individuals with a relatively low level of serotonin. In fact, this represents an intriguing topic for future research.

In critically reflecting on the current work, we want to acknowledge the fact that the study was of correlational nature. Thus, no causal conclusions can be drawn; we cannot say that cortisol inhibits costly punishment or that low cortisol leads to stronger costly punishment. Accordingly, this research remains silent concerning the exact causal mechanisms and the more specific bio-psychological processes involved in the observed relation. However, this more fine-grained level of analysis was not in the focus of the present research. Our aim was to investigate one basal endocrinological factor with respect to individuals’ tendency to engage in costly punishment. We also acknowledge the fact that our study involved exclusively male participants. It is an open question whether the relation of cortisol and costly punishment found can be generalized to women because there are remarkable differences in the activity of the HPA axis between the sexes [25], [75], [83], [84] which is also true for reactive aggression [85]. We further want to acknowledge that it is unclear whether the inverse relation of cortisol and costly punishment will also be found in different cultures and societies. This is particularly noteworthy given that costly punishment of uncooperative individuals differs in a remarkable way across cultures and societies [86], [87].

To conclude, the present work emphasizes that it is important to take endocrinological factors into account in order to comprehensively understand behavior in social dilemma situations. As such, the present work represents a promising approach to the study of sanctions that foster cooperation.

### Footnotes

^1^We used the term costly punishment instead of the commonly used term ‘altruistic punishment’ because it is potentially misleading to speak of altruistic punishment while arguing that costly punishment of uncooperative individuals reflects reactive aggression with the goal to harm another individual. These considerations are also in line with the finding that affective empathy, a trait that is consistently linked to prosocial, altruistic helping behavior [21], [22], was found to be negatively associated with costly punishment [23]. If costly punishment of uncooperative individuals reflected a prosocial, altruistic act it should correlate positively with other prosocial tendencies (such as affective empathy).

^2^In this work, we specifically focus on reactive aggression while we readily acknowledge the fact that there is an enormous amount of research involving other forms of aggression (e.g., proactive aggression, covert and overt aggression, or displaced aggression [41], [42]). Of note, findings regarding other forms of aggression and cortisol produced heterogeneous results [7], [9]–[11]. In this regard, given that the focus of the present contribution is on reactive aggression, a systematic elaboration of other forms of aggression would shift the focus of the manuscript. Thus, we refrain from a discussion of the relation between other forms of aggression and cortisol. Along these lines, we also want to note that whereas proactive aggression is explicitly conceptualized as an instrumental type of behavior this aspect is not explicitly mentioned in conceptualizations of reactive aggression. From our perspective, however, it is important to emphasize that reactive aggression can also serve an instrumental function. For instance, reactive aggression can be instrumentally used to defend oneself or one’s family [14].

^3^In another research report [37], we report on findings regarding the punishment of cooperative individuals (termed antisocial punishment [72]) based on the data obtained in the study we are reporting on in the present manuscript combined with a separate second study on antisocial punishment. In the present manuscript, we exclusively report on the findings referring to the punishment of uncooperative individuals (costly punishment). Accordingly, the two research reports are definitely not redundant and do not reflect duplicate publication. Nonetheless, we want to establish transparency regarding the fact that specific results of the present study are included in another research report.
